# Dose-Response Relationship between Serum Retinol Levels and Survival in Patients with Colorectal Cancer: Results from the DACHS Study

**DOI:** 10.3390/nu10040510

**Published:** 2018-04-19

**Authors:** Haifa Maalmi, Viola Walter, Lina Jansen, Robert W. Owen, Alexis Ulrich, Ben Schöttker, Jenny Chang-Claude, Michael Hoffmeister, Hermann Brenner

**Affiliations:** 1 Division of Clinical Epidemiology and Aging Research, German Cancer Research Center (DKFZ), Im Neuenheimer Feld 581, 69120 Heidelberg, Germany; h.maalmi@DKFZ.de (H.M.); v.walter@dkfz.de (V.W.); l.jansen@Dkfz.de (L.J.); b.schoettker@Dkfz.de (B.S.); m.hoffmeister@dkfz.de (M.H.); 2 Network Aging Research (NAR), Heidelberg University, Bergheimer Strasse 20, 69115 Heidelberg, Germany; 3 Medical Faculty Heidelberg, University of Heidelberg, 69120 Heidelberg, Germany; 4 Division of Preventive Oncology, German Cancer Research Center (DKFZ) and National Center for Tumor Diseases (NCT), Im Neuenheimer Feld 460, 69120 Heidelberg, Germany; Robert.Owen@nct-heidelberg.de; 5 Department of General, Visceral and Transplantation Surgery, University Hospital, Im Neuenheimer Feld 110, 69120 Heidelberg, Germany; alexis.ulrich@med.uni-heidelberg.de; 6 Division of Cancer Epidemiology, German Cancer Research Center (DKFZ), Im Neuenheimer Feld 581, 69120 Heidelberg, Germany; j.chang-claude@dkfz-heidelberg.de; 7 German Cancer Consortium, German Cancer Research Center (DKFZ), Im Neuenheimer Feld 280, 69120 Heidelberg, Germany

**Keywords:** colorectal neoplasms, mortality, vitamin A, vitamin D, dose-response relationship

## Abstract

Current knowledge on the role of retinol in the prognosis of patients with colorectal cancer (CRC) is very limited. We investigated the association of serum retinol levels with survival outcomes in a large cohort of 2908 CRC patients from Germany. Retinol concentrations were determined in serum collected shortly after diagnosis by mass spectrometry. Associations between serum retinol levels and survival outcomes were assessed using multivariable Cox regression and dose-response analyses. The joint association of serum retinol and serum 25-hydroxyvitamin D_3_ (25(OH)D_3_) with survival outcomes was also examined. During a median follow-up of 4.8 years, 787 deaths occurred, 573 of which were due to CRC. Dose-response curves showed an inverse relationship between serum retinol levels and survival endpoints in the range of <2.4 µmol/L, but no associations at higher levels. Low (<1.2 µmol/L) versus high (≥2.4 µmol/L) serum retinol levels were associated with poorer overall survival (Hazard ratio (HR) = 1.46, 95% confidence interval (CI) = 1.19–1.78, *P-trend* = 0.0003) and CRC-specific survival (HR = 1.69, 95% CI = 1.33–2.15, *P-trend* < 0.0001). Joint presence of low serum retinol (<1.2 µmol/L) and low 25(OH)D_3_ (<30 nmol/L) was associated with a particularly strong decrease in overall and CRC-specific survival. Low serum retinol levels were identified as a predictor of poor survival in CRC patients, in particular when co-occurring with low serum concentrations of 25(OH)D_3_. The clinical implications of these findings require further investigation.

## 1. Introduction

Colorectal cancer (CRC) is the fourth most common cause of cancer mortality worldwide [1]. Therefore, in patients with established disease, the ascertainment of factors that may ameliorate their prognosis is highly needed.

Vitamin A (retinol) is a nutrient obtained from the diet as either preformed vitamin A (retinol, retinyl esters) derived from animal products or provitamin A carotenoids (α-carotene, β-carotene, and β-cryptoxanthin) in colored vegetables [2]. In the cells, retinol is enzymatically oxidized to all-*trans* retinal and then to retinoic acid (RA) that exists in the form of two isomers called all-*trans* RA and 9-*cis* RA which are further transported to the nucleus to exert their physiological actions [3]. Retinol is supposed to have a role in inhibiting CRC progression. Suggested mechanisms include decreasing signaling via the major pathways that promote cell invasion and proliferation such as the β-catenin pathway [4], increasing tumor cell differentiation, and promoting tumor cell apoptosis [4,5,6,7,8]. In addition, retinoic acid (RA) plays critical roles in modulating inflammation throughout the intestine. Recently, Penny et al. showed that altered RA metabolism contributes to inflammation and tumorigenesis in mice, which is attenuated after restoration of RA concentrations by pharmacological blockade of the RA-catabolizing enzyme CYP26A1 [9]. In the epidemiological level, studies have shown an association between low serum retinol levels and decreased survival rate [10,11,12]. However, evidence regarding different survival outcomes and dose-response relationships has been limited due to the small numbers of studies and patients included.

Vitamin A belongs to the family of fat-soluble vitamins that includes vitamin D. However, beyond their biochemical similarity and their common anti-cancer properties [4,13], the two nutrients have similar molecular mechanisms of action. Their respective active forms (retinoid acids (RA) and 1α,25-dihydroxyvitamin D_3_ (1,25(OH)_2_D_3_)) and nuclear receptors (retinoic X receptor (RXR) and vitamin D receptor (VDR)) must dimerize with the RXR in order to be functional which may lead to a possible interaction between the two nutrients [14].

Therefore, the primary aim of this study was to investigate the association between serum retinol levels and various survival outcomes (overall, CRC-specific, recurrence-free and disease-free survival) in a large cohort of CRC patients. The secondary aim was to examine the joint association of serum retinol and serum 25-hydroxyvitamin D_3_ (25(OH)D_3_) levels, the best established marker for vitamin D status, with various survival outcomes.

## 2. Materials and Methods

### 2.1. Study Design and Study Population

The study population for this analysis included patients diagnosed with CRC and recruited in the DACHS study (**Da**rmkrebs: **Ch**ancen der Verhütung durch **S**creening), an ongoing multi-center population-based case-control study with additional active follow-up of cases conducted in the Rhine-Neckar region in the South of Germany. Details of the DACHS study have been reported elsewhere [15,16,17,18,19]. Briefly, patients with a first diagnosis of CRC aged 30 years or older were recruited in the 22 hospitals providing first-line therapy for CRC in the study region (approximately 2 million inhabitants). The study was approved by the ethics committees of the University of Heidelberg and the state medical boards of Baden-Württemberg and Rhineland-Palatinate, and written informed consent was obtained from each participant.

### 2.2. Data Collection

At recruitment either during hospital stay or shortly afterwards at the patients’ homes, detailed information on medical history, sociodemographic and lifestyle factors was collected by standardized questionnaires in personal interviews of approximately one hour conducted by trained interviewers. In addition, blood samples were drawn and stored at −80 °C. Detailed clinical data on patient and tumor characteristics were extracted from medical records obtained from hospitals and the physicians in charge of care after hospital discharge.

### 2.3. Follow-Up

Recruited patients were followed up with respect to therapy, the course of the disease, and survival. Briefly, the vital status of participants was determined through population registries at 3 and 5 years after CRC diagnosis, and death certificates were obtained from health authorities to determine the cause of death. Approximately three years after diagnosis information on CRC treatment and recurrence was collected from the patients’ attending physicians using a standardized questionnaire. About 5 years after diagnosis, further information about medical, lifestyle and recurrence history was collected through a standardized follow-up questionnaire filled out by the patients. If the patient died before the follow-up or did not complete the follow-up questionnaire, information on cancer recurrence before death was collected from the last attending physicians.

### 2.4. Outcomes

Follow-up time for overall, CRC-specific, recurrence-free and disease-free survival endpoints was calculated in days starting from the date of CRC diagnosis and ending at the date of having the event (death from any cause, death from CRC, recurrence or death from CRC, recurrence or death from any cause, respectively). Patients not reaching a specific endpoint were censored at a point in time when they were last known to have been alive or free of recurrence. In analyses of CRC-specific and recurrence-free survival, patients dying from causes other than CRC were censored at the time of their death.

### 2.5. Laboratory Measurements

High-Performance Liquid Chromatography-Electro Spray Ionization-Mass Spectrometry (HPLC-ESI-MS) in positive-ion mode was used to measure retinol and 25(OH)D_3_ in 70 µL of serum. All measurements were conducted blind with respect to outcomes in the Division of Preventive Oncology at the German Cancer Research Center over a period of 6 months. The HPLC-ESI-MS method was standardized using a pooled human serum, purchased from a local blood bank, and the Standard Reference Material (SRM) 972a developed by the National Institute of Standards and Technology (NIST) [20].

### 2.6. Covariates Assessment

Demographic, lifestyle and medical factors, known or suspected to be associated with serum retinol concentrations or CRC prognosis were extracted from patient questionnaires and medical records including: sex, age, month of blood draw, cancer stage at diagnosis, history of diabetes, history of hypertension, history of cardiovascular diseases (heart failure, myocardial infarction, angina pectoris, and stroke), smoking, alcohol consumption, physical activity, and consumption of red meat, fish, milk, vegetables, salad, and fruits.

### 2.7. Statistical Analysis

From 3146 patients recruited between 2003 and 2010, a total number of 2908 patients were included in this analysis after excluding 234 patients with missing serum, 2 patients with missing retinol measurement as well as 2 patients lost to follow-up.

Characteristics of the study population were analyzed using descriptive statistics. Cox proportional hazards models were used to estimate hazard ratios (HRs) and 95% confidence intervals (CIs) with respect to various survival endpoints. Different sets of adjustment variables were defined a priori. In model 1, analyses were adjusted for sex (male, female), age (continuous) and the month of blood draw. In model 2, analyses were additionally adjusted for cancer stage at diagnosis (I–IV according to the International Union Against Cancer (UICC) classification), history of diabetes (yes, no), history of hypertension (yes, no), history of cardiovascular diseases (heart failure, myocardial infarction, angina pectoris, and stroke) (yes, no), smoking (never, former, current), physical activity (tertiles of Metabolic Equivalent of Task hours per week (MET-h/week) in the last 12 months; low < 80.6, moderate 81 < 146.5, high 146.5+), alcohol consumption (none, high, low; commonly used sex-specific definitions, women: cut-off = 16 g ethanol/day; men: cut-off = 24 g ethanol/day), red meat and fish consumption [high: >once/week; low: ≤once/week], and milk, vegetables, salad, and fruit consumption [high: ≥once/day; moderate: once/week to <once/day; and low: <once/week]. In model 3, analyses were adjusted for covariates of model 2 and additionally for serum 25(OH)D_3_. In all analyses, we excluded participants with missing covariate data (exclusions <4.8% in all analyses) and performed the survival analyses with complete cases. Since the participants were enrolled into the study at varying time intervals after CRC diagnosis, we adjusted all survival analyses for late entry in days. The proportional hazards assumption was tested using interaction terms of covariates with time. Potential interactions between covariates and retinol levels were investigated by adding pertinent product terms to the full model and evaluating the corresponding Wald tests.

Adjusted survival curves were used to visualize patients’ survival according to serum retinol quintiles [21]. Dose-response relationships between serum retinol levels and survival outcomes were plotted with restricted cubic splines using predefined knots at the upper limits of retinol quintiles 1 to 4 and using the upper limit of the 4th quintile as the reference [22]. The correlation between serum retinol and serum 25(OH)D_3_ levels was analyzed graphically and by the Spearman rank correlation coefficient. Due to the skewed distribution of both nutrients, the log transformation was used. Cox regression models with the same covariates described for model 2 above were used for the analyses addressing the joint associations of low serum retinol and low serum 25(OH)D_3_ with the various survival outcomes. All statistical tests were 2-sided with an α level of 0.05, and all analyses were carried out using SAS statistical software (version 9.3; SAS Institute, Inc., Cary, NC, USA).

## 3. Results

### 3.1. Patient Characteristics

Socio-demographic, clinical, and dietary characteristics of the 2908 participants included in this study are shown in Table 1. The study population included more men (60%) than women (40%). Median age was 69 years (range: 30–96). Approximately, half of the patients were diagnosed in early stage (I or II, 53%) or late stage (III or IV, 47%), and serum retinol levels showed a strong inverse relationship with stage at diagnosis. Less than half of the patients (46%) had never smoked, approximately one third (30%) reported that they never drank alcohol. With 52% and 19%, respectively, histories of hypertension and diabetes were common. Almost three out of four participants (72%) reported consumption of red meat more than once per week. By contrast, fish consumption more than once per week was relatively rare (17%). Only 16% and 14% of patients reported consumption of vegetables or salad at least once a day, respectively.

### 3.2. Serum Concentrations of Retinol and 25(OH)D_3_

In adults, serum retinol concentrations are highly regulated in the human body within a range of 1–3 µmol/L. Therefore, serum retinol concentrations <1 µmol/L are considered deficient and >3 µmol/L are considered high [23,24,25]. In the current study, 397 (14%) of patients had low retinol levels, 1772 (61%) had retinol levels within the normal range, and 739 (25%) had high retinol levels. The median level (interquartile range) of serum retinol was 2.0 µmol/L (1.3–3.0 µmol/L). According to the Institute of Medicine’s definition, serum 25(OH)D_3_ levels below 30 nmol/L are considered to reflect vitamin D deficiency [26]. In our study population, the prevalence of vitamin D deficiency was very high (59%), and the median level (interquartile range) of serum 25(OH)D_3_ was 25.2 nmol/L (13.9–40.8 nmol/L). Mean retinol levels were much lower among patients with vitamin D deficiency (2.1 µmol/L) than among patients with insufficient (2.6 µmol/L) or sufficient vitamin D levels (2.8 µmol/L) (Table 1).

### 3.3. Serum Retinol Levels and Survival

During a median follow-up period of 4.8 years for overall survival and 3.9 years for recurrence-free survival, a total of 787 deaths occurred, of which 573 were due to CRC, and 817 patients recurred.

**Figure 1:**
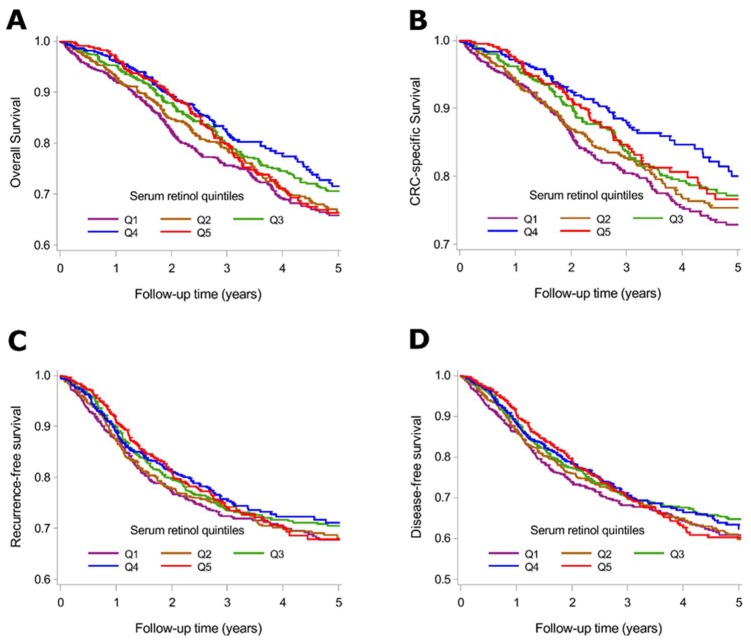
Adjusted survival curves for overall survival (**A**); CRC-specific survival (**B**); recurrence-free survival (**C**); and disease-free survival (**D**) according to serum retinol quintiles. Survival curves were adjusted for: sex, age at diagnosis, month of blood draw, cancer stage at diagnosis, history of diabetes, history of cardiovascular diseases, history of hypertension, smoking, alcohol consumption, physical activity, meat, fish, milk, vegetables, salad, and fruit consumption, serum 25-hydroxyvitamin D_3_ and late entry. Abbreviations: CRC: Colorectal cancer; Q: Quintile.

Figure 1 shows the adjusted survival curves according to serum retinol quintiles. Compared to CRC patients in the highest serum retinol quintiles, those in the lowest serum retinol quintiles had a lower survival rate.

**Figure 2:**
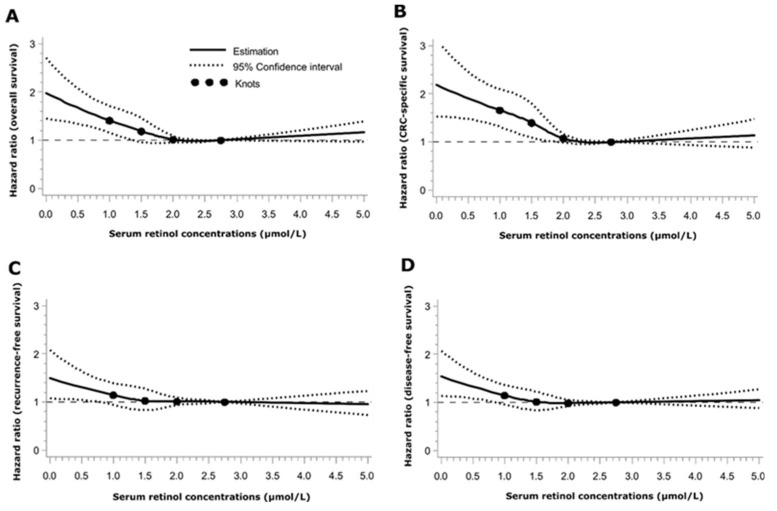
Dose-response relationship plots between serum retinol levels and overall survival (**A**); CRC-specific survival (**B**); recurrence-free survival (**C**); and disease-free survival (**D**). All plots were adjusted for: sex, age at diagnosis, month of blood draw, cancer stage at diagnosis, history of diabetes, history of cardiovascular diseases, history of hypertension, smoking, alcohol consumption, physical activity, meat, fish, milk, vegetables, salad, and fruit consumption, serum 25-hydroxyvitamin D_3_ and late entry. Point estimates of HRs (bold line) and 95% CIs (dotted lines) were obtained by fit of restricted cubic splines with knots at the upper limit of each serum retinol quintile: 1.2, 1.8, 2.4 and 3.3 µmol/L. The value 3.3 was set as the reference. Abbreviations: CRC: Colorectal cancer.

Restricted cubic spline curves yielded an inverse dose-response relationship between serum retinol levels and survival endpoints in the range of <2.4 µmol/L, i.e., within the lower three quintiles of the retinol distribution. Higher serum retinol levels did not show any significant association with the survival outcomes (Figure 2).

Table 2 shows the adjusted hazard ratios (HRs) and 95% confidence intervals (CIs) for the various survival endpoints according to serum retinol quintiles. With adjustment for sex, age and month of blood draw, low serum retinol levels (quintile 1) compared to serum levels in quintiles 4 and 5 were significantly associated with decreased survival throughout all outcomes. Further comprehensive adjustment for multiple other covariates (model 2) attenuated these associations but significant inverse trends persisted for all survival outcomes. Stage was the main confounder responsible for attenuating these associations. When serum 25(OH)D_3_ was taken into account as an additional potential confounder, associations were further slightly attenuated, but a strong significant inverse pattern for overall and CRC-specific survival persisted (P-trend 0.0003 and <0.0001, respectively). Low (<1.2 µmol/L) versus high serum retinol levels (≥2.4 µmol/L) were associated with decreased overall (HR = 1.46, 95% CI = 1.19–1.78) and CRC-specific survival (HR = 1.69, 95% CI = 1.33–2.15).

### 3.4. Joint Asociations of Serum Retinol and 25(OH)D_3_ Levels with Survival Outcomes

Supplementary Figure S1 shows the scatter plot of the correlation between the logarithmic values of serum retinol and serum 25(OH)D_3_ levels. Both nutrients were positively correlated (r = 0.30, *p* < 0.0001). Table 3 shows the joint association of serum retinol and serum 25(OH)D_3_ with survival outcomes. No association with survival was observed for high serum retinol concentrations (≥2.4 µmol/L) in the presence of vitamin D deficiency. However, strong and significantly decreased overall and CRC specific survival rates were seen among patients having both low serum retinol levels (<1.2 µmol/L) and vitamin D deficiency.

## 4. Discussion

To date, this is the largest prospective study investigating the association between circulating retinol and survival outcomes in CRC patients, and the first to evaluate the shape of this association. We found that lower serum retinol levels were associated with decreased overall and CRC-specific survival even after comprehensive adjustment for serum 25(OH)D_3_ and other covariates. Associations with recurrence-free and disease-free survival were weaker and not statistically significant in analyses with retinol quintiles. However, dose-response curves showed significant associations with these outcomes at very low retinol levels <1 µmol/L. Dose-response curves showed that survival gradually decreased for serum retinol levels falling below approximately 2.4 µmol/L. Moreover, our study found particularly strong decreases in survival in the presence of both low serum retinol (<1.2 µmol/L) and vitamin D deficiency (25(OH)D_3_ < 30 nmol/L).

### 4.1. Magnitude of Retinol Deficiency

Serum retinol is a common surrogate biochemical measure to evaluate vitamin A deficiency in the human body. Serum retinol depends on dietary intake of vitamin A in form of provitamin A carotenoids (mainly β-carotene) or preformed vitamin A (e.g., retinyl esters) [27]. Since in developed countries, intake of preformed vitamin A often exceeds the recommended dietary allowances (RDA) [25], very low serum retinol concentrations are uncommon, and may be detected only in individuals with poor nutritional vitamin A intake. In this study, low retinol concentrations (<1 µmol/L) were only observed in 14% of the participants which may reflect their inadequate nutrients intake. However, apart from poor dietary intake, cancer patients may also have their serum retinol levels lowered as a consequence of inflammation [10,11,28,29] which increases with advanced disease stages.

### 4.2. Retinol Status and CRC Survival

Colorectal cancer survival has been rarely studied in relation to circulating retinol, with only three small studies published to date [10,11,12]. In accordance with our results, Melichar et al. [10] found low serum concentrations of retinol, measured in blood samples collected prior to treatment, to be predictive of poor survival. However, this finding was limited by the very small sample size (25 CRC patients with metastatic disease) and the use and report of only median survival comparisons. Similarly, a positive association with survival was observed by Cooney et al. in a larger study including 368 CRC patients [11]. This significant association of higher vs. lowest retinol levels with improved survival remained consistent even after adjustment for C-reactive protein, a major inflammatory marker known to be associated with decreased retinol concentrations in cancer patients [30,31]. Likewise, in a study of only 53 CRC patients with liver metastases, lower CRC-specific survival was found in patients with plasma retinol concentrations below the median (<1 µmol/L) compared to those with plasma retinol concentrations above the median [12]. Despite the emerging evidence for low retinol concentrations being associated with CRC prognosis, further research based on large scale epidemiological and intervention studies is required to corroborate the results and assess potential underlying mechanisms. In addition to retinol, other vitamin A compounds such as provitamin A carotenoids were investigated in CRC. But available evidence does not support an association with survival [11,12].

### 4.3. Associations with CRC Prognosis Compared to Other Health-Related Outcomes

While available knowledge on the role of blood retinol concentrations in CRC prognosis remains sparse due to the small number of studies, several prospective cohort studies have examined potential associations between blood retinol concentrations, carotenoid intake and/or blood concentrations and a number of chronic diseases. A harmful effect of high retinol concentrations was found for, but not limited to, bone-related diseases [32,33].

Results of the Α-Tocopherol Βeta-Carotene (ATBC) cancer prevention trial from Finland [34] and the Beta-Carotene and Retinol Efficacy Trial (CARET) from the USA [35], showing an increased risk of lung cancer (by 16% and 28% in the two trials respectively) in the groups taking β-carotene supplements, raised concern about the effect of high retinol on health. In the absence of evidence, it was suggested that the effects of retinoid may be outcome-dependent.

### 4.4. Chemopreventive Role of Retinoid and Vitamin D

The association of vitamin A and vitamin D with cancer survival likely involves different biological mechanisms. The two nutrients have an effect on multiple cancer hallmarks, including reducing cancer cell proliferation, angiogenesis, invasion, and inflammation, as well as stimulating cancer cell differentiation and apoptosis [4,36,37]. Beyond these direct biological effects, both nutrients influence CRC pathogenesis indirectly through their ability to involve the RXR that is known for its role in decreasing signaling via the β-catenin pathway, a major pathway that promotes CRC progression [38]. In this large study, we found prognosis of CRC patients to be particularly poor in the presence of both low retinol concentrations and low 25(OH)D_3_ concentrations.

### 4.5. Strengths and Limitations

The strengths of this study include the prospective design with a long follow-up, the large number of participants, the use of HPLC-ESI-MS as the gold standard technique to measure both serum 25(OH)D_3_ and serum retinol, the ascertainment of different survival endpoints with a comprehensive adjustment for a large number of potential confounding factors, and the detailed dose-response analyses. In addition, this study is the first to investigate the joint association of low serum concentrations of retinol and 25(OH)D_3_ with long-term survival of CRC patients. However, some limitations of this study should also be addressed. First, despite careful adjustment for a large number of potential confounders, the observational design of the study implies the possibility of residual confounding and does not allow for conclusions on causality. Second, only baseline concentrations of serum retinol and serum 25(OH)D_3_ could be assessed which imperfectly reflect the long-term levels of these nutrients that are subject to change over the time-course of treatment. Furthermore, our investigation regarding the role of vitamin A in CRC prognosis was restricted to retinol and we did not consider other vitamin A compounds, such as retinyl esters and β-carotene. Studies investigating bone-related outcomes found contradictory results when retinol and β-carotene were assessed as exposures [33,39]. In addition, retinol levels are homeostatically controlled in the human body until liver reserves become dangerously low, which is why measurement of serum retinol is a sensitive method for determining vitamin A status only when levels are largely depleted or extremely high [40]. Therefore, future studies investigating the vitamin A status in association with different health-related outcomes should make use of new methods, such as isotope dilution assays, to trace total body reserves of vitamin A [40].

## 5. Conclusions

This study provides strong evidence that low serum retinol concentrations are a predictor of poor survival in CRC patients, in particular when co-occurring with low 25(OH)D_3_.

## Figures and Tables

**Table 1:** Socio-demographic, clinical and dietary characteristics of the study population (N = 2908).

		N (%)	Serum Retinol [µmol/L]	
Characteristics			Mean (SD)	*p*-Value *
Sex	Men	1731 (60%)	2.4 (1.6)	0.0310
	Women	1177 (40%)	2.3 (1.3)	
Age at diagnosis (years)	30–59	579 (20%)	2.4 (1.4)	0.0470
	60–69	942 (32%)	2.3 (1.4)	
	70–79	949 (33%)	2.4 (1.7)	
	80+	438 (15%)	2.2 (1.3)	
Cancer stage at diagnosis (UICC)	I	641 (22%)	2.6 (1.6)	<0.0001
	II	893 (31%)	2.4 (1.7)	
	III	940 (32%)	2.3 (1.3)	
	IV	425 (15%)	1.9 (1.4)	
History of diabetes	Yes	536 (19%)	2.3 (1.5)	0.0013
	No	2353 (81%)	2.6 (1.5)	
History of hypertension	Yes	1480 (52%)	2.4 (1.7)	0.0006
	No	1392 (48%)	2.2 (1.2)	
History of cardiovascular diseases	Yes	721 (25%)	2.4 (1.5)	0.0737
	No	2181 (75%)	2.3 (1.5)	
Smoking	Never	1324 (46%)	2.3 (1.3)	0.0019
	Former	1138 (39%)	2.5 (1.8)	
	Current	440 (15%)	2.2 (1.3)	
Alcohol consumption ^1^	None	852 (30%)	2.3 (1.3)	0.1114
	Low	1286 (45%)	2.4 (1.4)	
	High	729 (25%)	2.3 (1.8)	
Physical activity ^2^	Low	950 (33%)	2.4 (1.5)	0.0767
	Moderate	949 (33%)	2.3 (1.7)	
	High	949 (33%)	2.3 (1.4)	
Red meat	High: *>once/week*	2081 (72%)	2.4 (1.6)	0.0643
	Low: *≤once/week*	822 (28%)	2.3 (1.3)	
Fish	High: >once/week	494 (17%)	2.4 (1.9)	0.1076
	Low: ≤once/week	2408 (83%)	2.3 (1.4)	
Milk	High: *≥once/day*	727 (25%)	2.4 (1.3)	0.1424
	Moderate: *≥once/week*	620 (22%)	2.3 (1.8)	
	Low: *<once/week*	1549 (53%)	2.3 (1.5)	
Vegetables	High: *≥once/day*	451 (16%)	2.2 (1.5)	0.0067
	Moderate: *≥once/week*	1986 (68%)	2.3 (1.3)	
	Low: *<once/week*	465 (16%)	2.5 (2.1)	
Salad	High: *≥once/day*	406 (14%)	2.6 (2.1)	0.0892
	Moderate: *≥once/week*	2389 (82%)	2.3 (1.4)	
	Low: *<once/week*	107 (4%)	2.1 (1.4)	
Fruit	High: *≥once/day*	1855 (64%)	2.4 (1.5)	0.0040
	Moderate: *≥once/week*	900 (31%)	2.3 (1.5)	
	Low: *<once/week*	141 (5%)	2.3 (1.6)	
Serum 25(OH)D_3_ (nmol/L) ^3^	Deficient (<30)	1725 (59%)	2.1 (1.5)	<0.0001
	Insufficient (30-<50)	721 (25%)	2.6 (1.7)	
	Sufficient (≥50)	462 (16%)	2.8 (1.5)	
Serum retinol (µmol/L)	Low (<1)	397 (14%)	0.7 (0.2)	<0.0001
	Normal (1–3)	1772 (61%)	1.9 (0.5)	
	High (>3)	739 (25%)	4.2 (1.6)	

Abbreviations: SD: Standard deviation; UICC: Union for International Cancer Control; 25(OH)D_3_: 25-hydroxyvitamin D_3_; ^1^ commonly used sex-specific definitions (women: cut off = 16 g ethanol/day; men: cut off = 24 g ethanol/day); ^2^ definitions according to metabolic equivalent task hours (Met-h)/week in the last 12 months categorized in tertiles (low < 80.6; moderate 81–146.5; high > 146.5); ^3^ cut-offs based on the American Institute of Medicine, variables with missing values: cancer stage at diagnosis (N = 9); history of diabetes (N = 19); history of hypertension (N = 36); history of cardiovascular diseases (heart failure, myocardial infarction, angina pectoris, and stroke) (N = 6); smoking (N = 6); current alcohol consumption in g ethanol/day (N = 41); physical activity (N = 60); red meat consumption (N = 5); fish consumption (N = 6); milk consumption (N = 12); vegetables consumption (N = 6); salad consumption (N = 6); fruit consumption (N = 12); Note: Missing values were excluded from percentage calculations; * Non-parametric Wilcoxon test for variables with 2 groups and Kruskal-Wallis test for variables with more than 2 groups.

**Table 2:** Association of serum retinol quintiles with overall, CRC-specific, recurrence-free and disease-free survival.

	Serum Retinol (µmol/L) by Quintiles				
	Quintile 1 (<1.2)	Quintile 2 (1.2 < 1.8)	Quintile 3 (1.8 ≤ 2.4)	Quintile 4–5 (≥2.4)	*p*-Trend
**Overall survival ^a^**					
No. at risk	534	542	550	1102	
No. of events	214	151	135	219	
Model 1, HR (95% CI) *	1.92 (1.60–2.31)	1.31 (1.07–1.60)	1.10 (0.89–1.36)	Reference	<0.0001
Model 2, HR (95% CI) **	1.56 (1.28–1.90)	1.30 (1.05–1.61)	1.04 (0.84–1.30)	Reference	<0.0001
Model 3, HR (95% CI) ***	1.46 (1.19–1.78)	1.25 (1.01–1.55)	1.03 (0.82–1.28)	Reference	0.0003
**CRC-specific survival ^a^**					
No. at risk	534	542	550	1102	
No. of events	169	114	100	139	
Model 1, HR (95% CI) *	2.44 (1.96–3.03)	1.54 (1.21–1.96)	1.35 (1.05–1.73)	Reference	<0.0001
Model 2, HR (95% CI) **	1.80 (1.43–2.28)	1.54 (1.20–1.99)	1.23 (0.94–1.60)	Reference	<0.0001
Model 3, HR (95% CI) ***	1.69 (1.33–2.15)	1.48 (1.15–1.92)	1.21 (0.93–1.58)	Reference	<0.0001
**Recurrence-free survival ^b^**					
No. at risk	520	523	537	1067	
No. of events	187	142	132	218	
Model 1, HR (95% CI) *	1.72 (1.42–2.08)	1.26 (1.02–1.55)	1.20 (0.97–1.48)	Reference	<0.0001
Model 2, HR (95% CI) **	1.25 (1.02–1.54)	1.15 (0.92–1.43)	1.08 (0.86–1.34)	Reference	0.0265
Model 3, HR (95% CI) ***	1.20 (0.97–1.47)	1.12 (0.90–1.39)	1.07 (0.86–1.33)	Reference	0.0790
**Disease-free survival ^b^**					
No. at risk	520	523	537	1067	
No. of events	229	176	162	282	
Model 1, HR (95% CI) *	1.60 (1.35–1.89)	1.19 (0.99–1.43)	1.09 (0.90–1.32)	Reference	<0.0001
Model 2, HR (95% CI) **	1.25 (1.04–1.49)	1.11 (0.91–1.34)	1.01 (0.83–1.23)	Reference	0.0188
Model 3, HR (95% CI) ***	1.18 (0.98–1.42)	1.07 (0.88–1.30)	1.00 (0.82–1.22)	Reference	0.0865

Abbreviations: HR: Hazard ratio; CI: Confidence interval; CRC: Colorectal cancer. Note: quintile 4 (2.4 ≤ 3.3) and quintile 5 (≥3.3) were grouped together to build the reference category. * Adjusted for: sex, age at diagnosis in years and month of blood draw. ** Additionally adjusted for: cancer stage at diagnosis (I–IV according to the International Union Against Cancer classification), history of diabetes (yes/no), history of cardiovascular diseases: heart failure, myocardial infarction, angina pectoris, and stroke (yes/no), history of hypertension (yes/no), smoking (never, former, current), alcohol consumption [none/low/high; commonly used sex-specific definitions; women: cut off = 16 g ethanol/day; men: cut off = 24 g ethanol/day)]; physical activity [definitions according to metabolic equivalent task hours (Met-h)/week in the last 12 months (low < 80.6; moderate 81–146.5; high > 146.5)]; red meat and fish consumption [high: >once/week; low: ≤once/week], milk, vegetables, salad, and fruit consumption [high: ≥once/day; moderate: ≥once/week; and low: <once/week] and time between diagnosis and recruitment in days *** Additionally adjusted for: serum 25(OH)D_3_. ^a^ Complete case analysis used 2728 participants (6% of patients had missing values); ^b^ Complete case analysis used 2647 participants (9% of patients had missing values).

**Table 3:** Joint associations of serum 25(OH)D_3_ and retinol levels with overall, CRC-specific, recurrence-free and disease-free survival.

	Serum 25(OH)D_3_, nmol/L	Serum Retinol Levels, µmol/L					
		<1.2		1.2 ≤ 2.4		≥2.4	
		N at risk/N Events	HR (95% CI)	N at Risk/N Events	HR (95% CI)	N at Risk/N Events	HR (95% CI)
**Overall survival ^a^**	<30	386/170	1.76 (1.23–2.50)	675/198	1.31 (0.93–1.85)	543/120	1.09 (0.75–1.57)
	30 ≤ 50	101/30	1.15 (0.71–1.85)	265/54	1.09 (0.72–1.65)	315/54	0.90 (0.59–1.36)
	≥50	38/13	1.65 (0.87–3.13)	168/39	0.96 (0.61–1.50)	237/41	**Reference**
**CRC-specific survival ^a^**	<30	386/132	2.06 (1.32–3.21)	675/148	1.55 (1.00–2.40)	543/76	1.12 (0.70–1.78)
	30 ≤ 50	101/24	1.31 (0.74–2.32)	265/42	1.46 (0.88–2.41)	315/34	0.86 (0.50–1.45)
	≥50	38/12	2.10 (1.02–4.31)	168/29	1.11 (0.64–1.91)	237/25	**Reference**
**Recurrence-free survival ^b^**	<30	376/149	1.29 (0.89–1.86)	655/191	1.07 (0.75–1.53)	526/119	0.92 (0.63–1.34)
	30 ≤ 50	98/25	0.67 (0.40–1.12)	255/50	0.89 (0.58–1.36)	304/57	0.76 (0.50–1.16)
	≥50	37/12	1.24 (0.64–2.42)	165/37	0.84 (0.53–1.33)	231/39	**Reference**
**Disease-free survival ^b^**	<30	376/185	1.25 (0.91–1.72)	655/237	1.02 (0.75–1.38)	526/154	0.90 (0.65–1.24)
	30 ≤ 50	98/30	0.67 (0.42–1.05)	255/60	0.77 (0.53–1.11)	304/71	0.74 (0.52–1.06)
	≥50	37/13	1.07 (0.57–1.99)	165/45	0.76 (0.51–1.13)	231/54	**Reference**

Abbreviations: HR: Hazard ratio; CI: Confidence interval; CRC: Colorectal cancer; 25(OH)D_3_: 25-hydroxyvitamin D_3_. All analyses were adjusted for: sex, age at diagnosis in years; month of blood draw; cancer stage at diagnosis (I–IV according to the International Union Against Cancer classification), history of diabetes (yes/no), history of cardiovascular diseases: heart failure, myocardial infarction, angina pectoris, and stroke (yes/no), history of hypertension (yes/no), smoking (never, former, current), alcohol consumption [none/low/high; commonly used sex-specific definitions; women: cut off = 16 g ethanol/day; men: cut off = 24 g ethanol/day)]; physical activity [definitions according to metabolic equivalent task hours (Met-h)/week in the last 12 months categorized in tertiles (low < 80.6; moderate 81–146.5; high > 146.5)]; red meat and fish consumption [high: >once/week; low: ≤once/week], milk, vegetables, salad, and fruit consumption [high: ≥once/day; moderate: ≥once/week; and low: <once/week] and time between diagnosis and recruitment in days. ^a^ Complete case analysis used 2728 participants (6% of patients had missing values); ^b^ Complete case analysis used 2647 participants (9% of patients had missing values).

## Supplementary Materials

The following are available online at http://www.mdpi.com/2072-6643/10/4/510/s1, Figure S1: Scatterplot with 95% confidence ellipse for log 25(OH)D_3_ vs log retinol.
