# Supplementary figures and images for: Dose-Response Relationship between Serum Retinol Levels and Survival in Patients with Colorectal Cancer: Results from the DACHS Study
Source: Nutrients. 2018 Apr 19;10(4):510. doi: 10.3390/nu10040510 (PMC5946295; doi:10.3390/nu10040510)

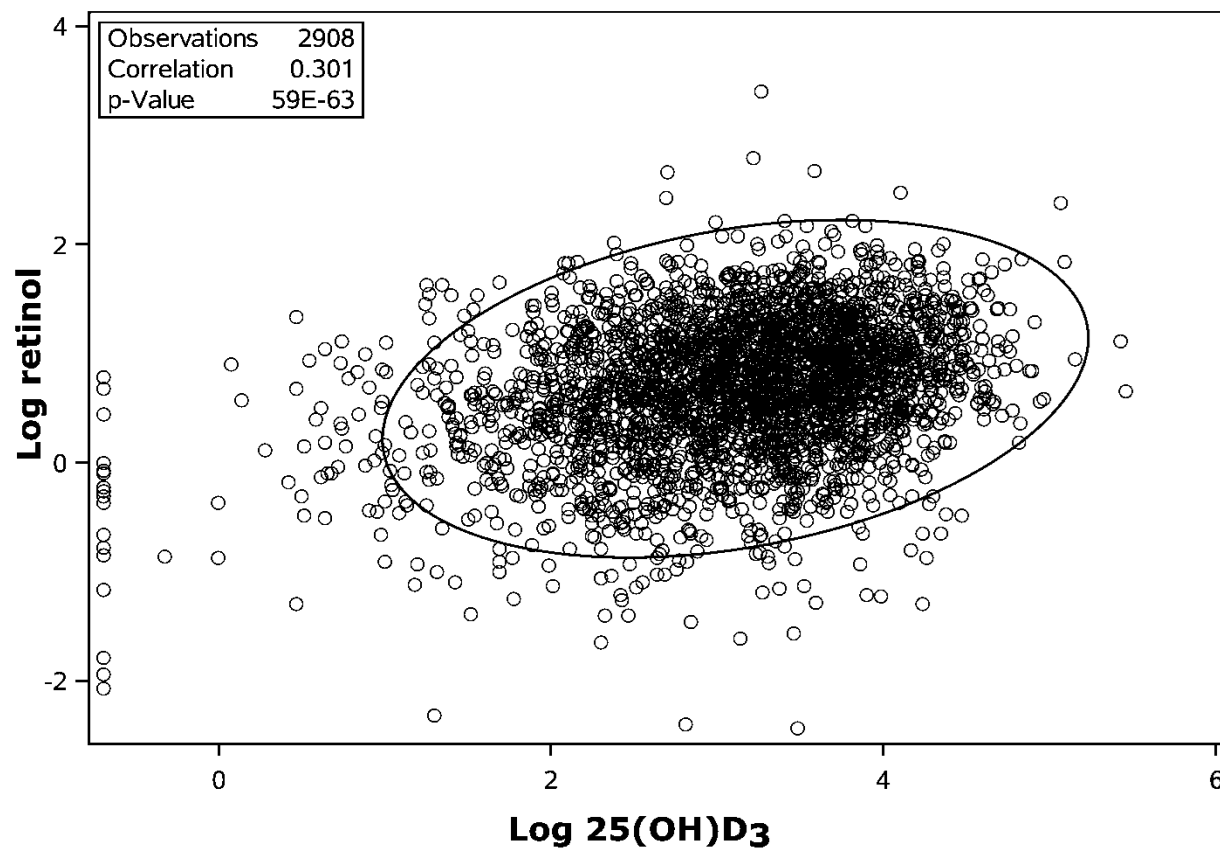

**Supplementary Figure 1:** Scatterplot with 95% confidence ellipse for log 25(OH)D<sub>3</sub> vs log retinol

Supplement: Supplementary file 1 [file nutrients-10-00510-s001.pdf]
